# Computed tomography-negative symptomatic intracerebral hemorrhage in a patient with cerebral small vessel disease: A case report

**DOI:** 10.1097/MD.0000000000021382

**Published:** 2020-07-17

**Authors:** Jinhee Han, Harin Yang, Jeong Hoon Bae, Hyun Young Kim, Young Seo Kim

**Affiliations:** Department of Neurology, College of Medicine, Hanyang University, Seoul, Republic of Korea.

**Keywords:** cerebral hemorrhage, computed tomography, microbleed, small vessel disease

## Abstract

**Rationale:**

Computed tomography plays a key role in the initial evaluation of suspected acute stroke by ruling out the possibility of hemorrhage before thrombolysis. Recently, many reports have described cases of symptomatic microbleeds, and there may also have been a case of computed tomography- negative intracerebral hemorrhage.

**Patient concerns:**

A 70-year-old female patient who had a history of lacunar infarction and severe small vessel disease developed dysarthria. On brain non-contrast computed tomography there was no evidence of intracerebral hemorrhage. However, brain magnetic resonance imaging performed at 3 hours after the initial computed tomography showed cerebral hemorrhage.

**Diagnoses:**

The diagnosis was computed tomography-negative intracerebral hemorrhage.

**Interventions:**

The patient was treated with cilostazole 100 mg twice a day with blood pressure management.

**Outcomes:**

The dysarthria was fully recovered within 5 days and the patient did not suffer recurrent stroke symptoms over the following 2 years.

**Lessons:**

In patients with underlying severe small vessel disease and microbleeds, there could be computed tomography-negative hemorrhage and susceptibility weighted magnetic resonance image could be needed. More attention is required before applying thrombolysis therapy because there is a possibility of cerebral hemorrhage in those patients.

## 1 Introduction

Stroke is an emergent disease which results in death and severe disability. However, in many cases it can be reversed by timely treatment such as intravenous thrombolysis and endovascular thrombectomy. Thus, prompt diagnosis and treatment are necessary to minimize stroke-induced disability, and brain imaging plays a major role in determining treatment strategy. Non-contrast brain computed tomography (NCCT) is commonly used to differentiate hemorrhage from ischemia because it takes a short time and has high sensitivity for detecting cerebral hemorrhage.^[1]^

Intravenous thrombolysis with tissue plasminogen activator (tPA) is first line treatment in ischemic stroke patients who present within 4.5 hours. However, hemorrhagic complications were found to develop in 21.3% of patients and symptomatic hemorrhage in 5.6%.^[2]^ Once hemorrhagic complications occur, neurologic symptom may deteriorate and patients may have poor outcomes. Thus, intravenous thrombolysis should only be undertaken after careful examination of early brain images for signs of potential hemorrhagic. The authors encountered a case of a patient with acute cerebral hemorrhage in whom early brain NCCT gave no evidence of bleeding but magnetic resonance imaging (MRI) indicated hemorrhage. Ruling out cerebral hemorrhage by brain CT may be insufficient, especially in patients with a history of severe small vessel disease and lacunar infarction. We describe this case with emphasis on the need for caution in employing intravenous thrombolysis in such patients.

## 2 Case report

**Figure 1:**
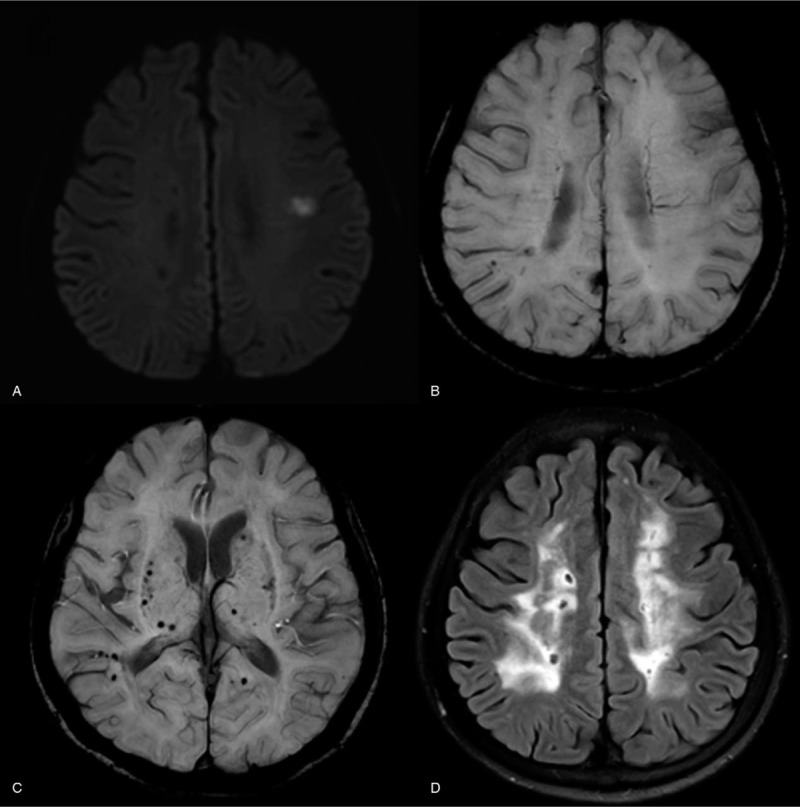
Brain magnetic resonance image obtained 8 months previously. Diffusion-weighted imaging (A) shows increased signal intensity in left corona radiata consistent with acute ischemic stroke. Susceptibility-weighted imaging(B) did not showed any hemorrhage in the ischemic stroke lesion. Susceptibility-weighted imaging reveals multiple micro-bleedings in the temporo-parietal cortex, thalamus and basal ganglia (C), and confluent white matter changes with multiple silent infarctions can be seen in the fluid-attenuated inversion recovery image (D), which implies underlying small vessel disease.

A 70-year-old woman visited the emergency room with dysarthria that had started 5 hours earlier. She had been diagnosed with hypertension 11 years previously and took regular medication. She had also been diagnosed with corona radiata infarction, severe white matter change and multiple microbleeds 8 months earlier and was taking 100 mg of cilostazole twice a day (Fig. 1). On physical examination, temperature was 36.8°C, heart rate 108/min and blood pressure 130/80 mm Hg. Laboratory findings such as complete blood count, electrolytes, liver function test, renal function test, and coagulation profiles were unremarkable. On neurologic examination she complained only of dysarthria, without hemiparesis or sensory change.

**Figure 2:**
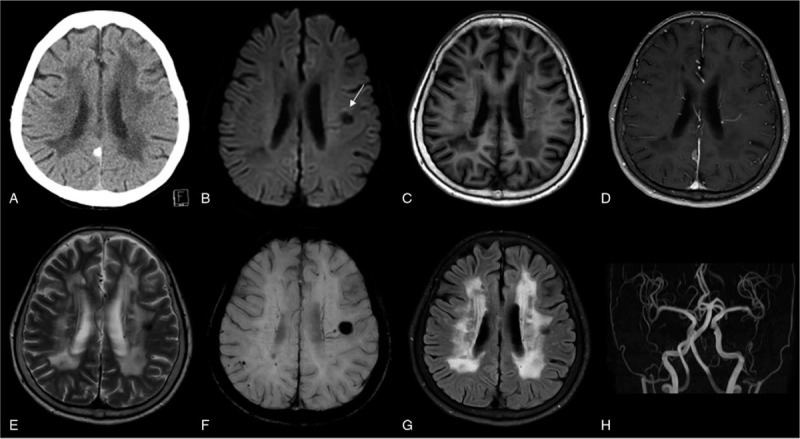
Brain computed tomography image obtained on the day of admission does not show any acute hemorrhage (A). Brain magnetic resonance image with contrast enhancement obtained 3 hours after the initial CT. The diffusion-weighted image shows decreased signal intensity in the left corona radiata and a perilesional high signal intensity towards the motor cortex (arrow) (B). T1 weighted image shows isointensity without enhancement (C, D). T2 weighted image (E) and Susceptibility-weighted imaging (F) show low signal intensity compatible with acute hemorrhage. The subcortical white matter changes seen in fluid-attenuated inversion recovery images were unchanged from the previous images (G). Brain magnetic resonance angiography shows no significant stenotic lesions in intracranial arteries (H).

Emergency brain CT, which was done in the emergency room, did not give evidence of cerebral hemorrhage (Fig. 2A). Since she had only mild symptoms and 5 hours had passed from symptom onset, she did not receive recombinant tissue plasminogen activator (rtPA), and antiplatelet agent was continued with conservative treatment. Brain MRI which was performed 3 hours after the CT showed a low signal intensity in the left corona radiata with a high signal rim on diffusion weighted image (DWI). T1 weighted image showed isointensity without enhancement, and T2 weighted image and susceptibility weighted image (SWI) showed low signal intensity compatible with acute stage cerebral hemorrhage (Fig. 2B-F). This was a new hemorrhage not evident on brain MRI taken 8 months before. Besides this, white matter changes and multiple silent brain infarctions were observed on fluid attenuated inversion recovery images that were unchanged from 8 months before (Fig. 2G). There was no stenotic lesion on brain magnetic resonance angiography (Fig. 2H). All this evidence together led us to suggest that the patient's dysarthria was caused by corona radiata hemorrhage.

The patient's symptoms gradually improved over 5 days of treatment, and cilostazole was maintained without change of medication. To determine the cause of the repeated stroke events, vasculitis-related factors, antiphospholipid syndrome screening, cerebral autosomal dominant arteriopathy with subcortical infarction genetic test and Fabry disease screening were performed with negative results. The patient was followed-up for 2 years in the outpatient clinic without recurrence of stroke symptoms.

## 3 Discussion

Non-contrast brain CT (NCCT) is the most useful mode of brain imaging for differentiating ischemic stroke from hemorrhagic stroke before deciding for or against thrombolysis. Hence, guidelines recommend NCCT for initial brain imaging for stroke patients.^[1]^ However, the occurrence of acute symptomatic cerebral hemorrhage not revealed by NCCT, as in this case, suggests that thrombolytic treatment based on NCCT alone may not be safe. Although this patient was not indicated for thrombolysis because of her mild symptoms and the delayed presentation, she was initially diagnosed as acute cerebral infarction and treatment was performed based on that diagnosis. However, DWI MRI performed 3 hours after CT did not reveal any restricted diffusion. In contrast, a relatively large low signal intensity was observed on SWI. If the patient had reached the emergency room within the time indicated for thrombolysis and had had more prominent neurologic symptoms, thrombolysis might have been carried out based on the results of NCCT and this might have caused a thrombolysis-associated hemorrhage. Although a recent meta-analysis concluded that cerebral microbleeds were not associated with symptomatic intracerebral hemorrhage (ICH) after intravenous thrombolysis, they were associated with poor functional outcome and various types of ICH.^[3]^ Thus, patients with ICH without a prominent high density on NCCT, as in this case, should be treated with great care.

There are 2 case reports of CT negative intracranial hemorrhage. In 1 case, a hypertensive thalamic hemorrhage was not detected by NCCT, but was observed by brain MRI taken 12 hours later.^[4]^ However, in that case there is a possibility of hemorrhagic transformation after acute cerebral infarction. In the other case, initial CT did not reveal a high density focus, but cerebral hemorrhage was observed by MRI taken 3 hours later.^[5]^ However, there was some disagreement about whether bleeding was really not evident on the CT scan, and it was suggested that bleeding can only be ruled out by close observation by NCCT. Although in our case cerebral hemorrhage occurred close to the previous cerebral infarction, the lesion had not been visible by MRI 8 months previously, and was consistent with the neurologic symptoms. Thus, we believe that it may be regarded as a newly developed cerebral hemorrhage. Recent reports emphasize that acute microbleeds which are not detected by brain CT may cause stroke symptoms.^[6–8]^ However, the bleeding area in our patient exceeded 5 mm, which is not within the range of microbleeds (2–5 mm), and so it may be regarded as a macrobleed or cerebral hemorrhage rather than a microbleed. One observational study has suggested that microbleeds are more likely to be symptoms of stroke when DWI shows a high signal rim around the low signal intensity.^[9]^ Our patient did have a high signal rim around the low signal lesion on DWI, and it was present in the motor cortex direction. Therefore, our patient's cerebral hemorrhage, which was invisible on NCCT, may have caused the stroke symptom.

Cerebral small vessel disease is produced by damaged brain arteries, arterioles, capillaries, and veins. It may lead to silent radiologic findings such as white matter changes, lacunar infarction, microbleeds, and enlarged Virchow Robin spaces. The pathologic changes include fibrinoid necrosis, lipohyalinosis, and Charcot Bouchard aneurysm, and the pathologic findings are the same as the findings of intracerebral hemorrhage.^[10]^ Thus, intracerebral hemorrhage is likely to occur in patients with severe small vessel disease and a higher burden of microbleeds.^[11]^ In addition, endothelial dysfunction, inflammation and rupture of microaneurysms can occur in small vessel disease patients, and may be associated with thrombolysis-related hemorrhage and poor functional outcome.^[12,13]^ In the present case, severe small vessel disease was confirmed by the past subcortical lacunar infarction, severe white matter changes on fluid attenuated inversion recovery images and multiple microbleeds on SWI. Although cerebral infarction with minor symptoms is not a reason for being excluded from thrombolysis and insisting on safety, careful consideration is needed before carrying out thrombolysis therapy in patients with severe small vessel disease because of the possibility of CT-negative hemorrhage, as in our case.

## Author contributions

JH drafted the manuscript. HY, JHB, HYK contributed to the intellectual discussion of the patient. YSK conceived to report the patient and drafted manuscript together. Each author has participated sufficiently in the work to take public responsibility for appropriate portions of the content.

**Conceptualization:** Young Seo Kim.

**Data curation:** Harin Yang, Jeong Hoon Bae, Young Seo Kim.

**Writing – original draft:** Jinhee Han.

**Writing – review & editing:** Young Seo Kim.
